# Supplementary material for: Quality control of protein synthesis in the early elongation stage
Source: Nat Commun. 2023 May 17;14:2704. doi: 10.1038/s41467-023-38077-5 (PMC10192219; doi:10.1038/s41467-023-38077-5)
Supplement: Supplementary file 1 — Supplementary Information [file 41467_2023_38077_MOESM1_ESM.pdf]

Supplemental information

Quality control of protein synthesis in the early elongation stage

Nagao et al.

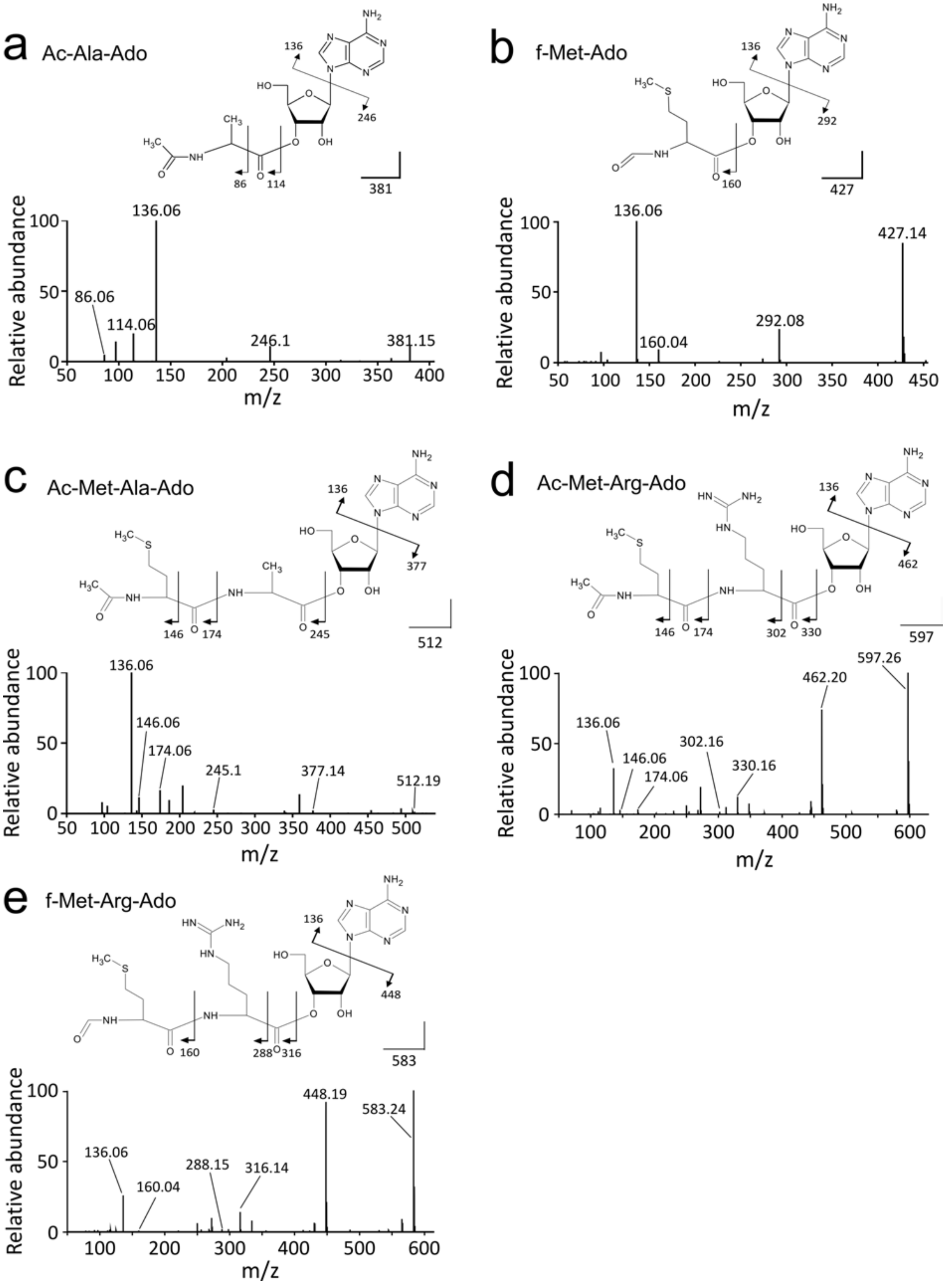

a
Ac-Ala-Ado
136
246
86
114
381
136.06
86.06
114.06
246.1
381.15
Relative abundance
m/z
b
f-Met-Ado
136
292
160
427
136.06
427.14
292.08
160.04
c
Ac-Met-Ala-Ado
136
377
146
174
245
512
136.06
146.06
174.06
245.1
377.14
512.19
d
Ac-Met-Arg-Ado
136
462
146
174
302
330
597
597.26
462.20
136.06
146.06
174.06
302.16
330.16
e
f-Met-Arg-Ado
136
448
160
288
316
583
448.19
583.24
136.06
160.04
288.15
316.14

f

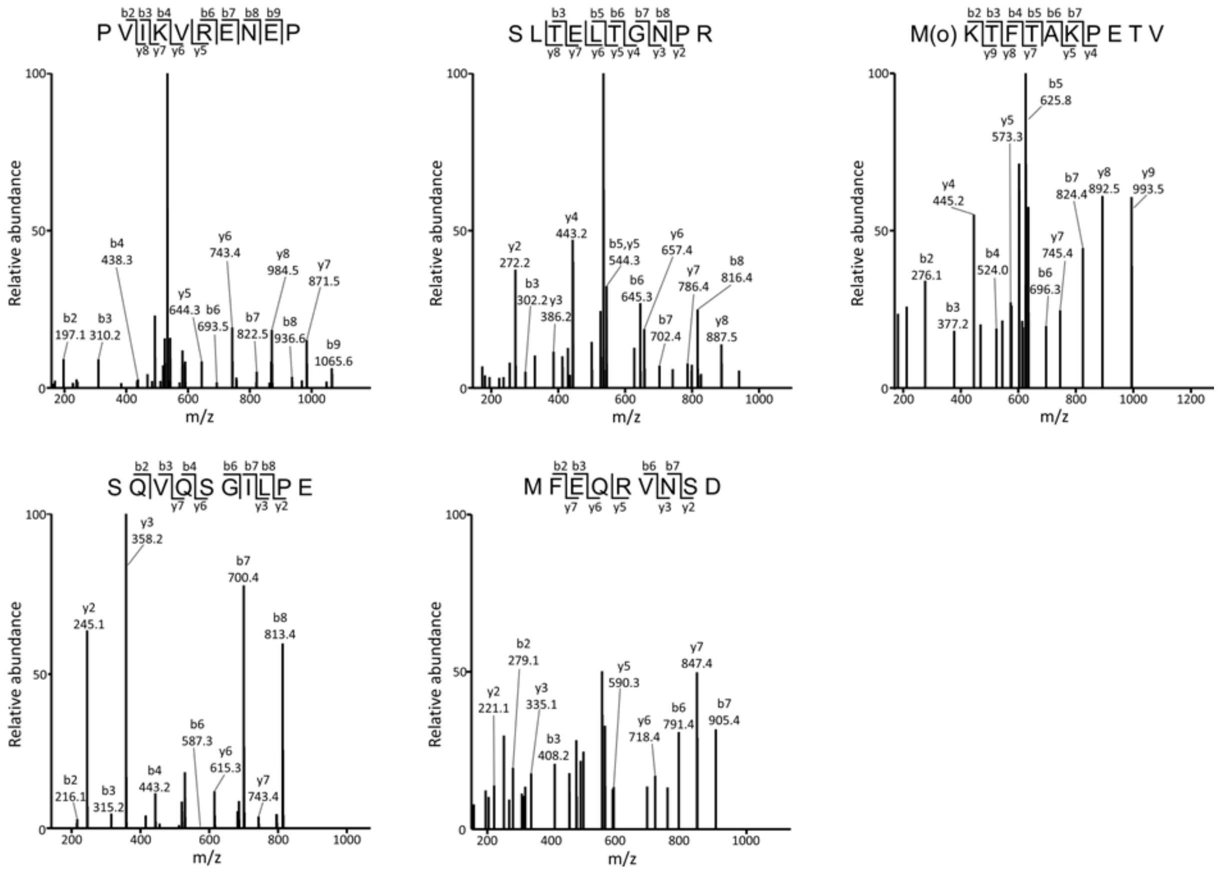

Supplementary Figure 1. Confirmation of chemical structures of Ac-aa-Ado, Ac-pep-Ado and nascent peptides of pep-tRNA profiling.

CID spectra of Ac-Ala-Ado **(a)**, f-Met-Ado **(b)**, Ac-Met-Ala-Ado **(c)**, Ac-Met-Arg-Ado **(d)**, f-Met-Arg-Ado **(e)** and nascent peptides **(f)**. MS/MS-based sequences of nascent peptides from pep-tRNAs. The product ions are assigned in the corresponding chemical structures and peptide sequences.

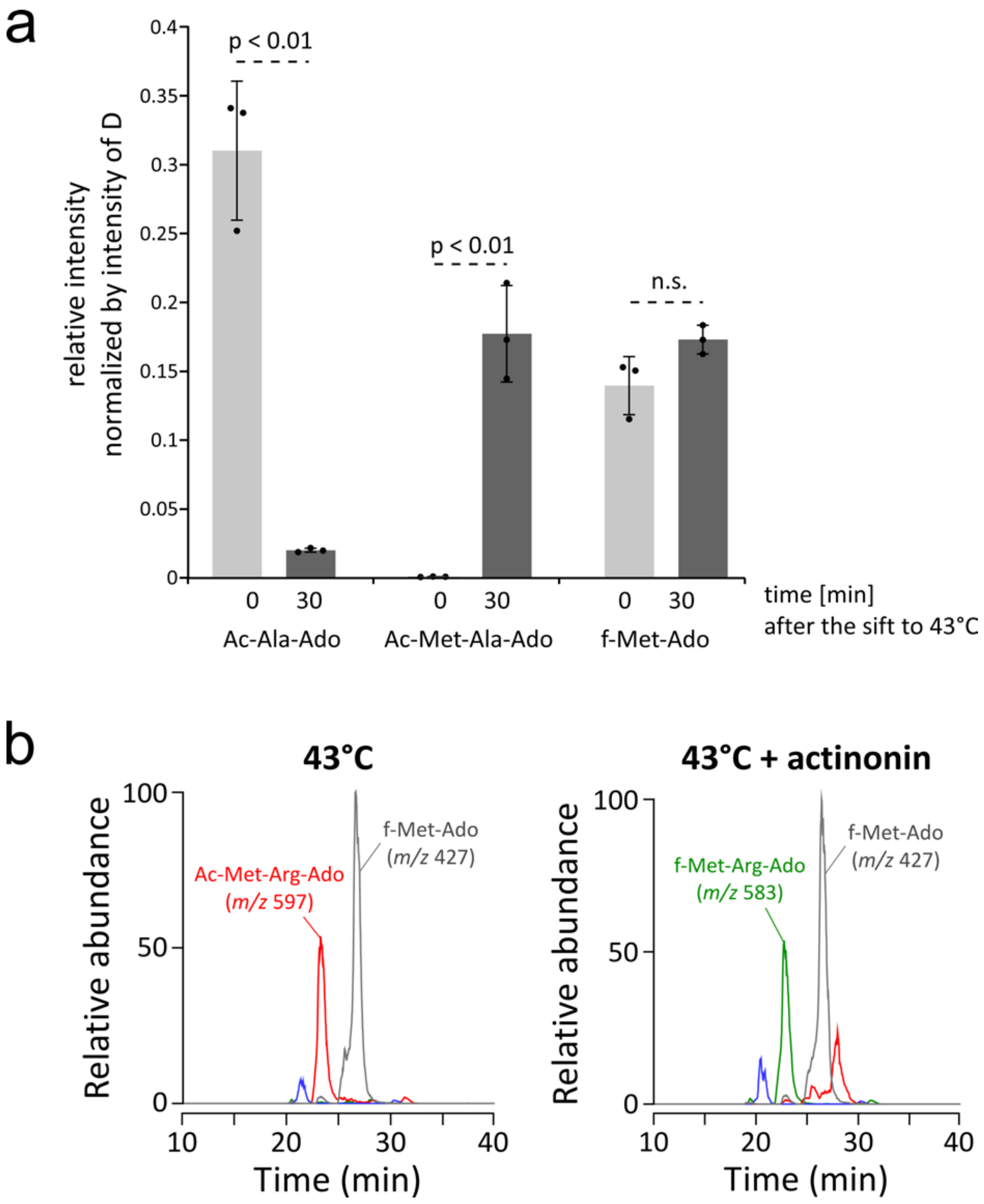

Supplementary Figure 2. LC/MS quantification of f-Met-Ado and Ac-dipep-Ado.

(a) Cellular level of f-Met-Ado and Ac-dipep-Ado. Relative intensity of Ac-Ala-Ado, Ac-Met-Ala-Ado and f-Met-Ado normalized by the intensity of dihydrouridine (D) in *pth*^{ts} cells at 0 min (light gray) and 30 min (gray) after shifting to 43°C. Data are presented as means ± s.d. of three independent experiments. $p<0.01$ (two-tailed *t*-test). Exact p-values for Ac-Ala-Ado and Ac-Met-Ala-Ado are 5.7×10^{-4} and 9.4×10^{-4}, respectively. Source data are provided as a Source

Data file.

(b) Formylated pep-tRNAs are efficiently deformylated by PDF. LC/MS nucleoside analyses of total tRNAs containing Ac-aa-tRNA and Ac-pep-tRNA prepared from the *ΔtolC*/*pth*^{ts} strain in logarithmic growth phase incubated at 43°C for 30 min in the absence (left panel) or presence (right panel) of actinonin. Mass chromatograms of Ac-Arg-Ado (*m/z* 466.21, blue), Ac-Met-Arg-Ado (*m/z* 597.26, red), f-Met-Arg-Ado (*m/z* 583.24, green), and f-Met-Ado (*m/z* 427.14, gray) are shown.

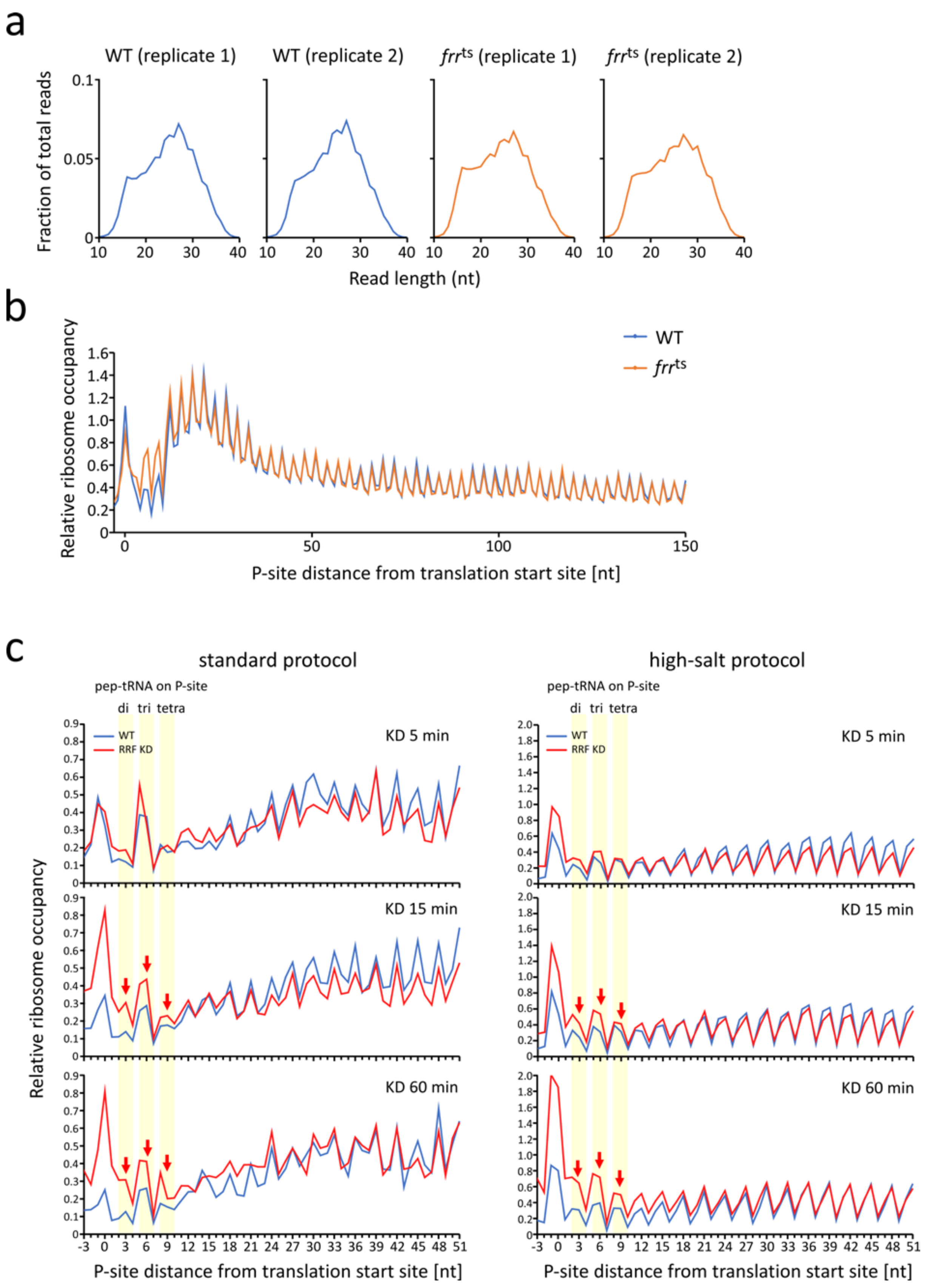

Supplementary Figure 3. Ribosome profiling of WT and *frr*^ts strains incubated at 43°C for

30 min.

(a) Distribution of read lengths of ribosome protected fragments (RPFs) in ribosome profiling of WT (blue) and *frr*^{ts} (orange). Each ribosome profiling analysis was performed in duplicate for both strains.

(b) Metagene analyses of RPFs mapped in the translation initiation regions up to 150 nt from the initiation site of *E. coli* ORFs for WT (blue) and *frr*^{ts} (orange) strains incubated at 43°C for 30 min.

(c) Metagene analyses of RPFs mapped around the translation initiation sites of *E. coli* ORFs for WT (blue) and RRF KD (red) strains. The data of ribosome profiling using a standard protocol (left panels) and with a high-salt lysis buffer (right panels) were obtained from the literature [1]. The time after RRF depletion and the peak positions that correspond di-, tri-, and tetra-pep-tRNAs on P-site are indicated in the figures.

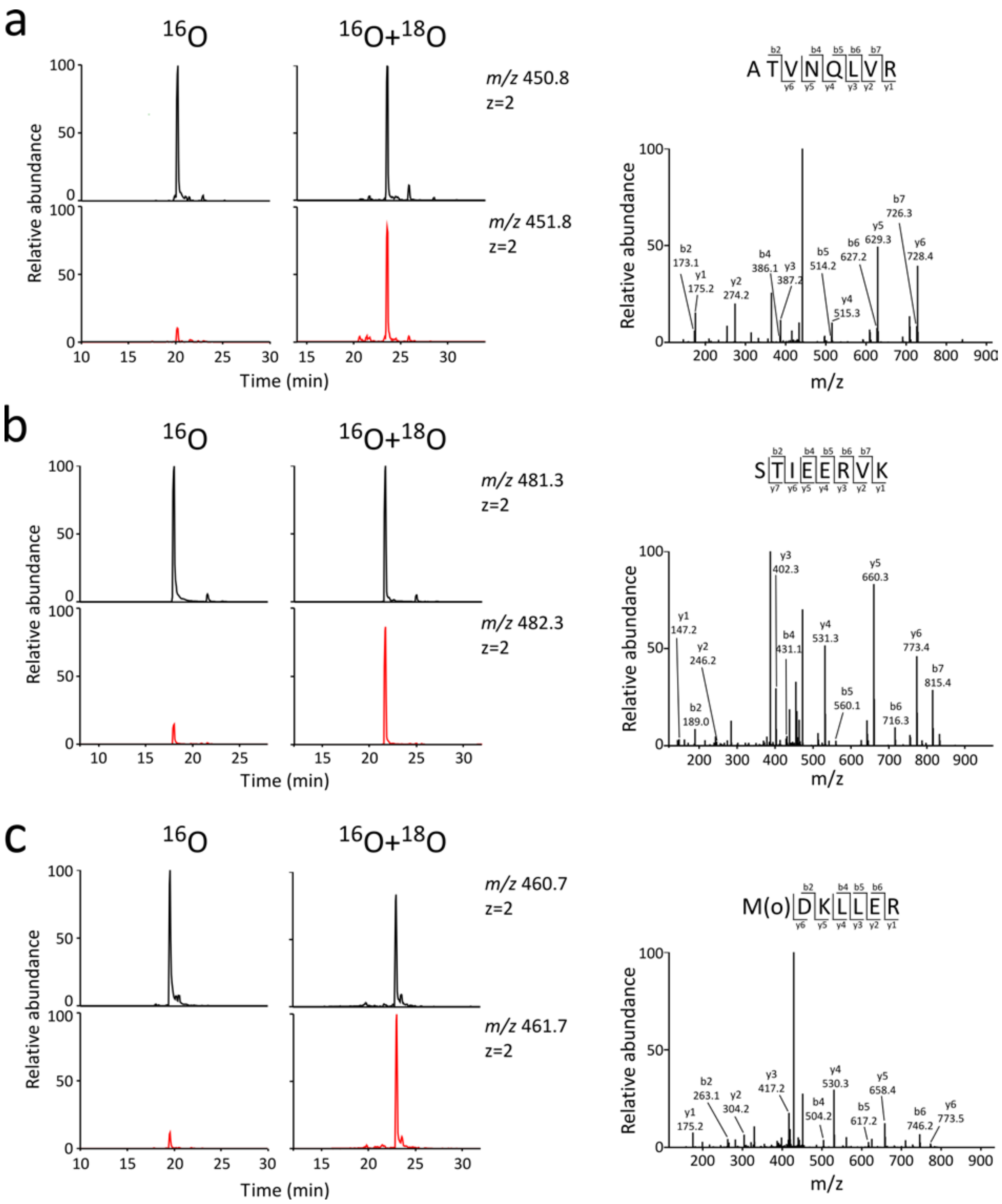

Supplementary Figure 4. Stable isotope labeling of C-termini of nascent peptides derived from pep-tRNAs.

(a–c) Total pep-tRNA fraction was hydrolyzed with normal water or 50% [^{18}O]-labeled water and subjected to LC/MS analyses. Mass chromatograms for doubly charged ions of three nascent peptides hydrolyzed with normal water (left panels) and 50% [^{18}O]-labeled water (right panels). Each peptide was sequenced by assigning the product ions of the CID spectrum (right-

most panels): ATVNQLVR from *rpsL* **(a)**, STIEERVK from *acpP* **(b)**, and M(o)DKLLER from *pepT* **(c)**. *m/z* and charge number for each peptide are indicated. M(o) stands for an oxidized Met.

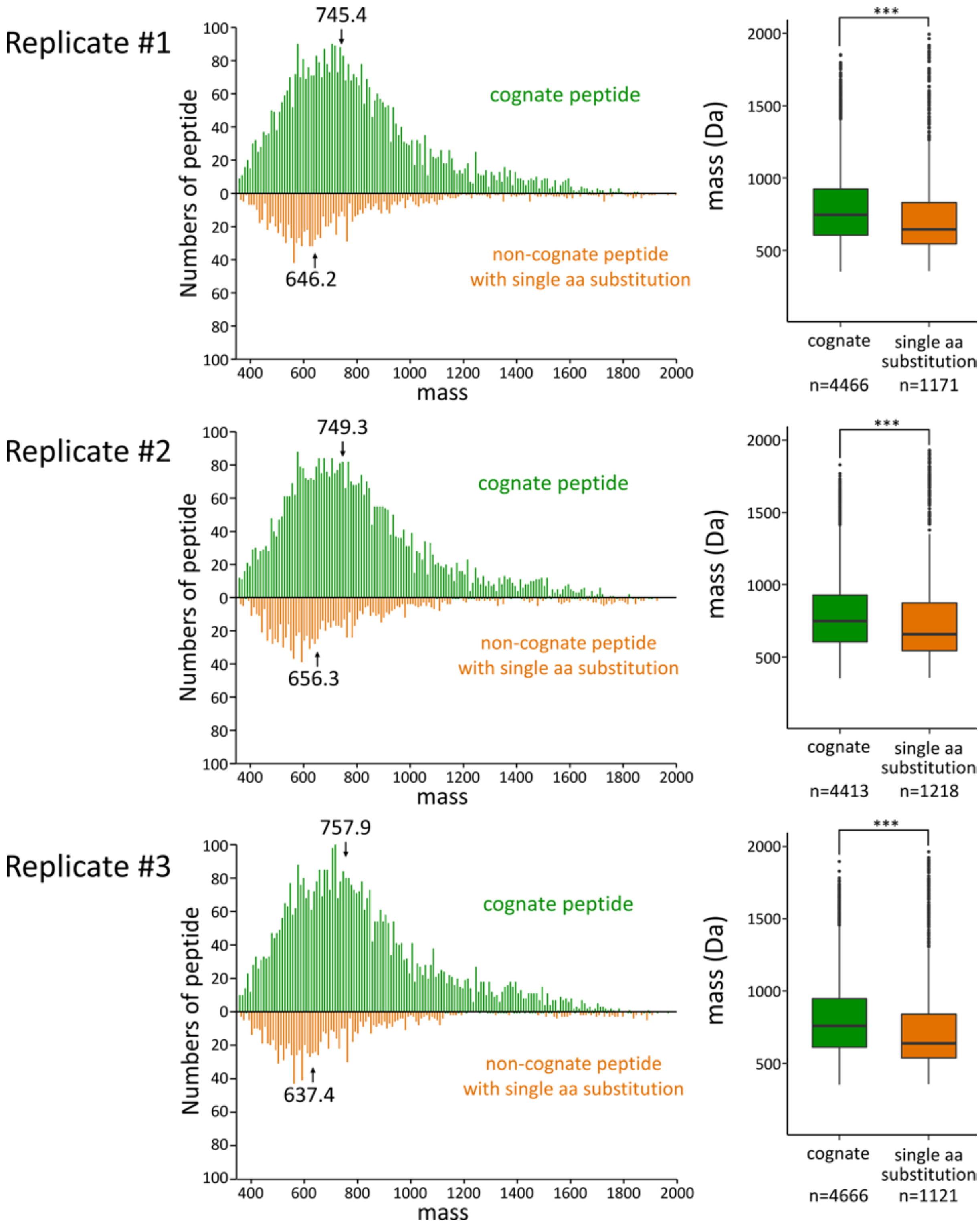

Supplementary Figure 5. Triplicate of pep-tRNA profiling in the *pth*^{ts} strain.

(Left panels) Histogram of peptide masses assigned to cognate (green) and non-cognate (orange) pep-tRNAs by pep-tRNA profiling for each replicate. Median masses of cognate and non-cognate peptides were indicated in each histogram. (Right panels) Boxplots of peptide masses assigned to cognate (green, n=4466, 4413 and 4666 for replicate #1, #2 and #3,

respectively) and non-cognate (orange, n=1171, 1218 and 1121 for replicate #1, #2 and #3, respectively) pep-tRNAs by pep-tRNA profiling for each replicate. Boxplots show median (central line), upper and lower quartiles (box limits), maximum and minimum (whiskers). ***$p<2.2\times10^{-16}$ (two-sided Wilcoxon rank–sum test).

Source data are provided as a Source Data file.

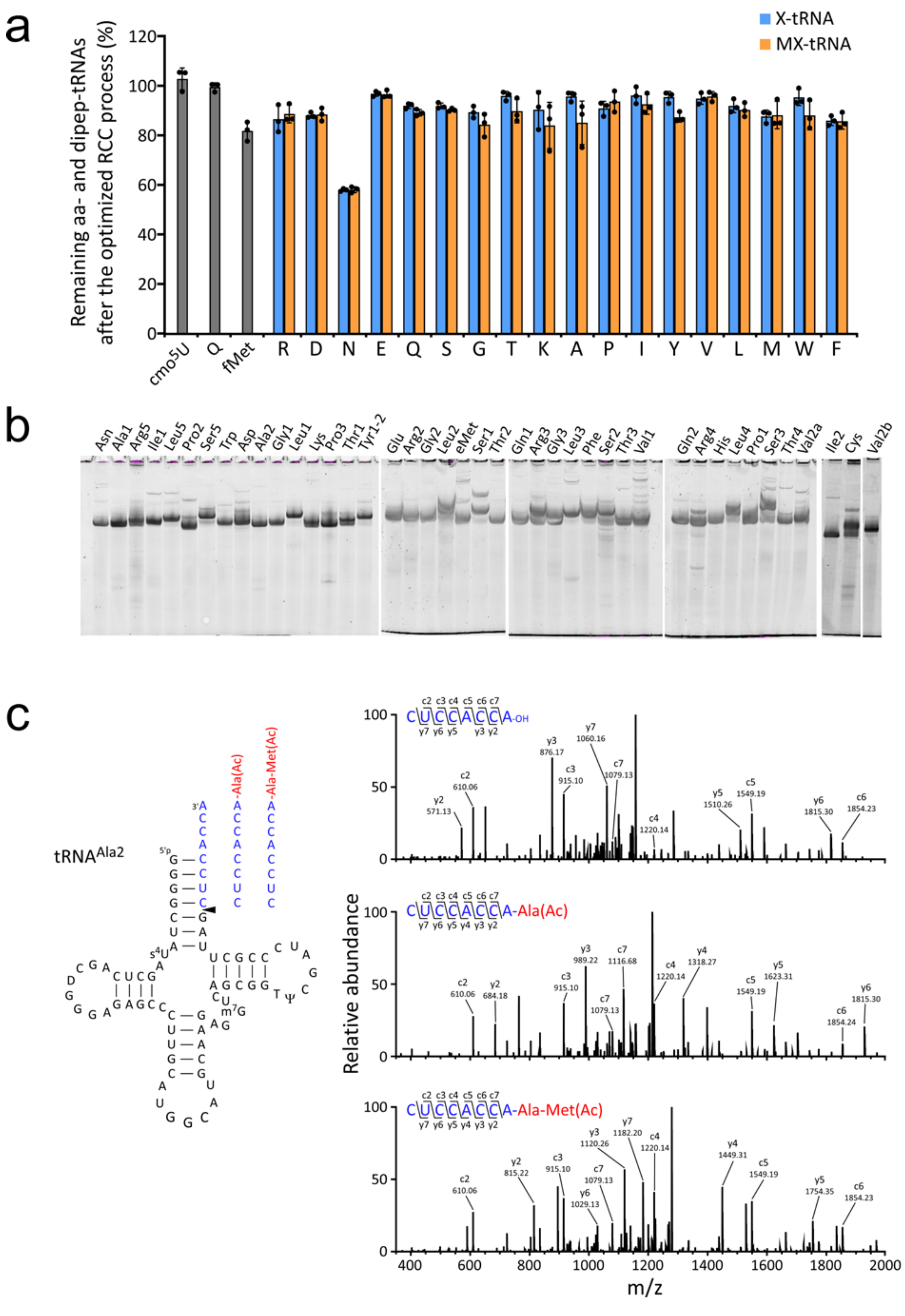
a
X-tRNA
MX-tRNA
Remaining aa- and dipep-tRNAs after the optimized RCC process (%)
0
20
40
60
80
100
120
cmo5U
Q
fMet
R D N E Q S G T K A P I Y V L M W F
b
Asn Ala1 Arg5 Ile1 Leu5 Pro2 Ser5 Trp Asp Ala2 Gly1 Leu1 Lys Pro3 Thr1 Tyr1-2
Glu Arg2 Gly2 Leu2 eMet Ser1 Thr2
Gln1 Arg3 Gly3 Leu3 Phe Ser2 Thr3 Val1
Gln2 Arg4 His Leu4 Pro1 Ser3 Thr4 Val2a
Ile2 Cys Val2b
c
tRNAAla2
-Ala(Ac)
-Ala-Met(Ac)
CUCCACCA-OH
CUCCACCA-Ala(Ac)
CUCCACCA-Ala-Met(Ac)
Relative abundance
m/z
400 600 800 1000 1200 1400 1600 1800 2000

Supplementary Figure 6. Isolation of individual pep-tRNA species.

(a) Remaining Ac-aa- (blue) and Ac-dipep-tRNAs (orange) treated with the same conditions according to the optimized RCC method. Sample treated with or without the RCC procedure was digested into nucleosides and subjected to LC/MS analyses. The remaining rate of each Ac-aa-Ado and Ac-dipep-Ado was calculated from the peak area of the corresponding mass chromatogram. Data are presented as means ± s.d. of three independent experiments. Two modified nucleosides, 5-carboxymethoxyuridine (cmo^5U) and queuosine (Q), are indicated as internal controls.

(b) The results of denaturing PAGE analyses of 42 individual pep-tRNA species isolated by the RCC method from the *pth*^{ts} cells incubated at 43°C for 30 min.

(c) RNA-MS of 3'-terminal fragments of the isolated pep-tRNA^{Ala2}. The left figure shows the secondary structure of *E. coli* tRNA^{Ala2} and its 3' terminus fragments acylated with Ac-Ala and Ac-Met-Ala generated by RNase T_1 digestion. The right panels show CID spectra of 3' terminal fragments with deacylation (top panel), Ac-Ala (middle panel), and Ac-Met-Ala (bottom panel). The product ions are assigned in the sequence of the corresponding fragment. Source data and unprocessed images are provided in Source Data file.

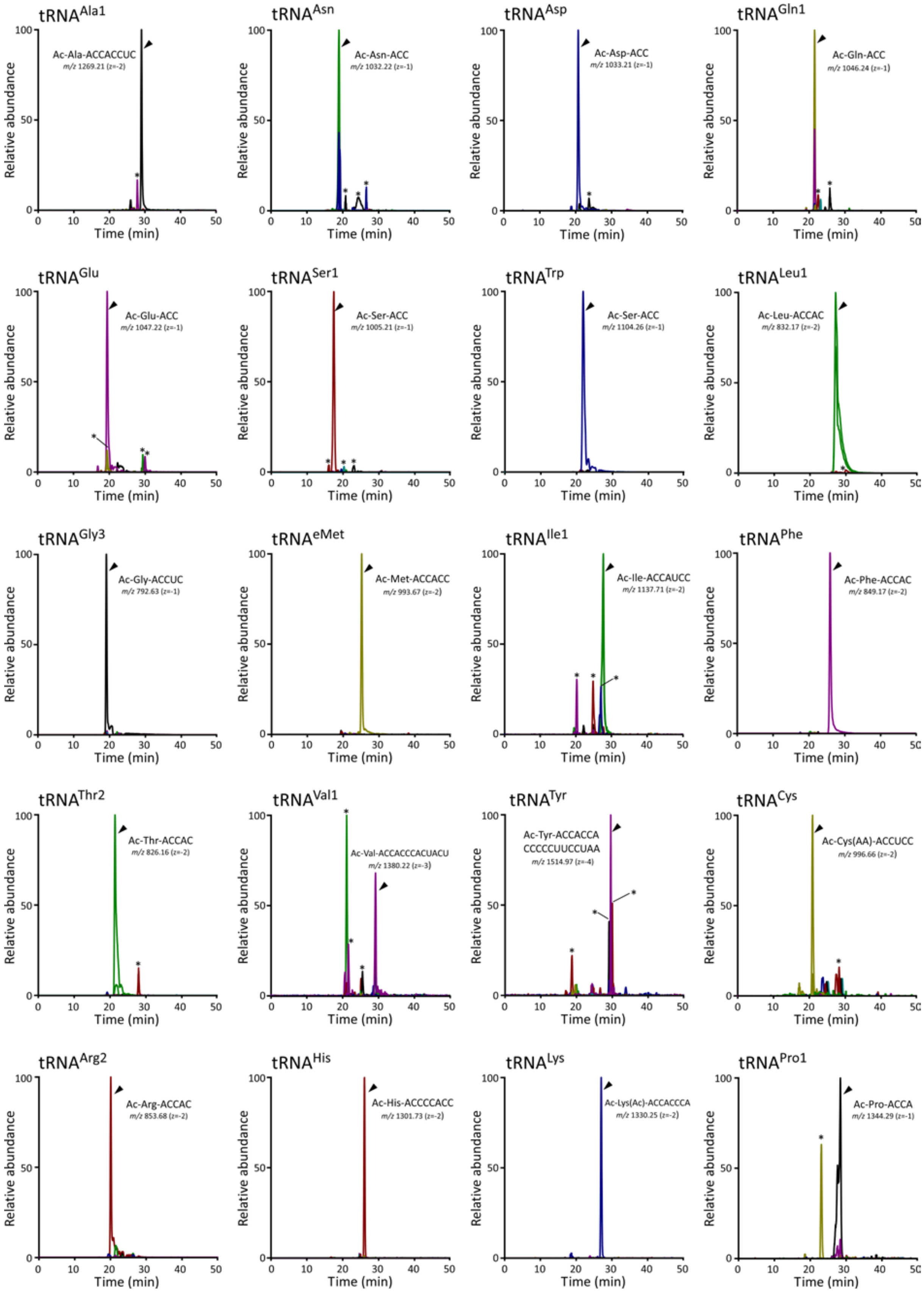

Supplementary Figure 7. RNA-MS of 3'-terminal fragments of isolated pep-tRNAs.

Twenty species of isolated pep-tRNAs were digested with RNase T_1 and subjected to RNA-MS to detect 3'-terminal fragments acylated with amino acids. The MS peak for each fragment acylated with its cognate amino acid is indicated by an arrowhead in each panel. The tRNA species, its sequence, and its *m/z* value are indicated in each panel. The peak indicated by * is non-specific.

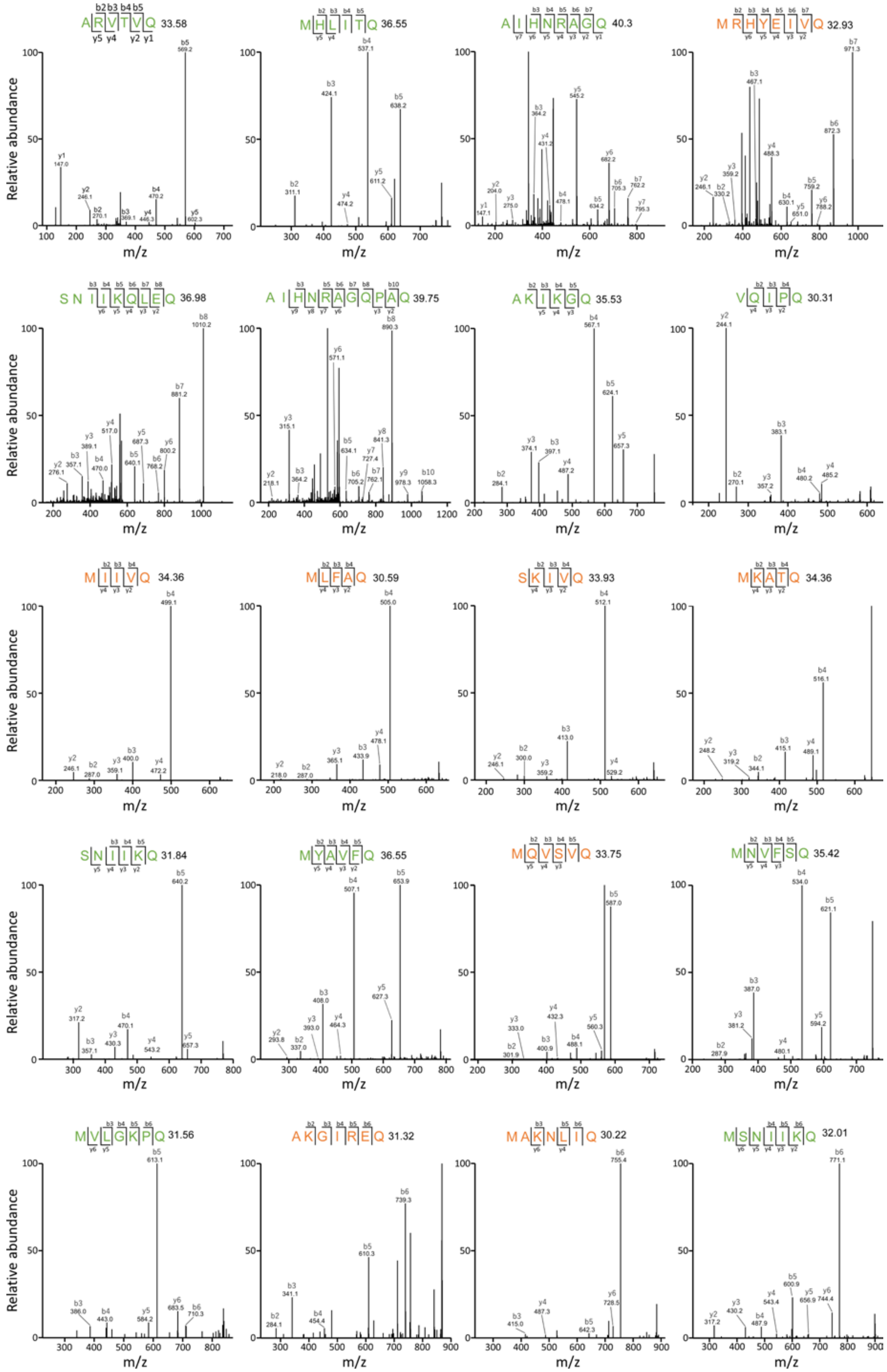

ARVTVQ 33.58
MHLITQ 36.55
AIHNRAGQ 40.3
MRHYEIVQ 32.93
SNIIKQLEQ 36.98
AIHNRAGQPAQ 39.75
AKIKGQ 35.53
VQIPQ 30.31
MIIVQ 34.36
MLFAQ 30.59
SKIVQ 33.93
MKATQ 34.36
SNIIKQ 31.84
MYAVFQ 36.55
MQVSVQ 33.75
MNVFSQ 35.42
MVLGKPQ 31.56
AKGIREQ 31.32
MAKNLIQ 30.22
MSNIIKQ 32.01
Relative abundance
m/z

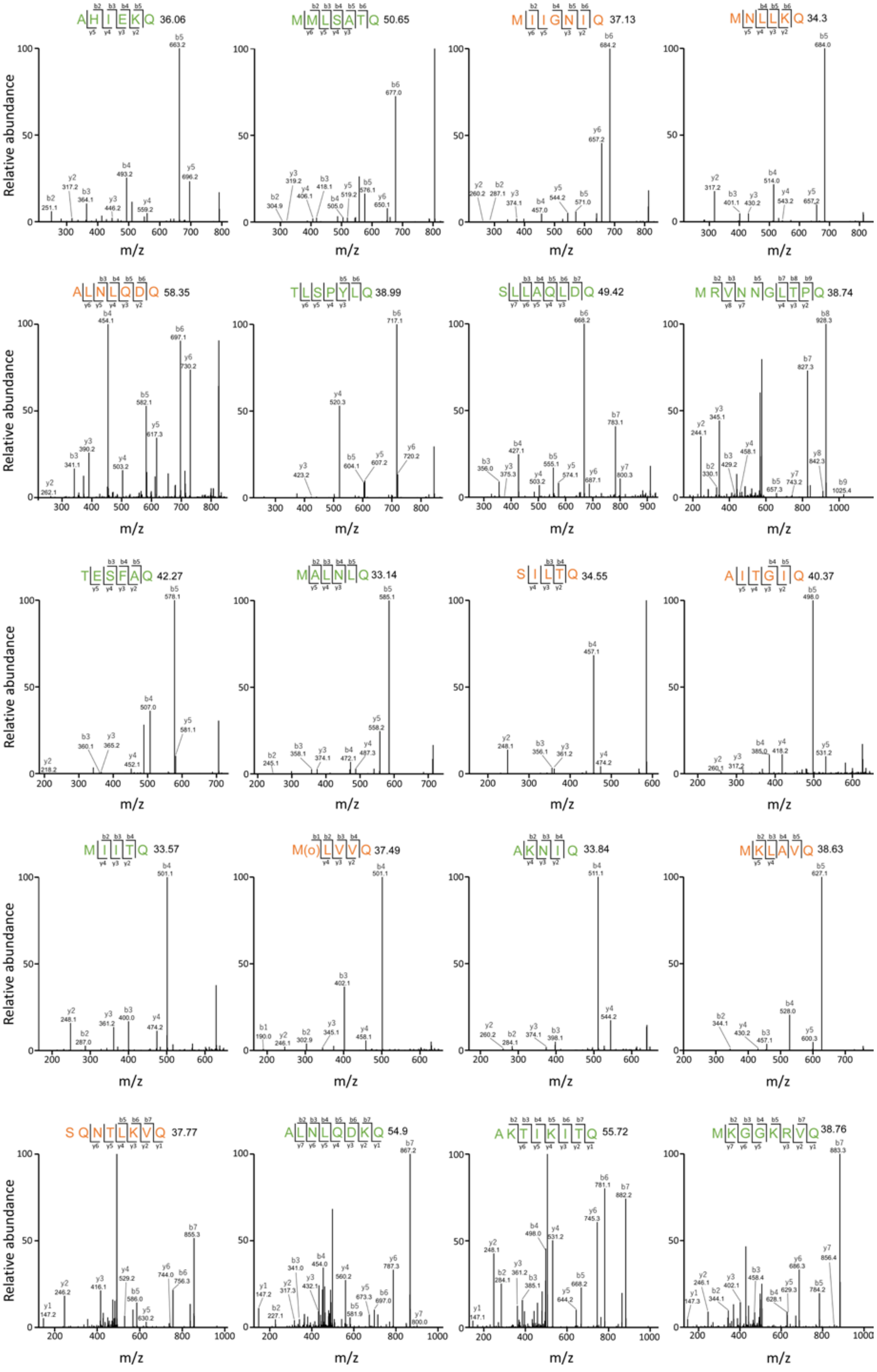

AHIEKQ 36.06
MMLSATQ 50.65
MIIGNIQ 37.13
MNLLKQ 34.3
ALNLQDQ 58.35
TLSPYLQ 38.99
SLLAQLDQ 49.42
MRVNNGLTPQ 38.74
TESFAQ 42.27
MALNLQ 33.14
SILTQ 34.55
AITGIQ 40.37
MIITQ 33.57
M(o)LVVQ 37.49
AKNIQ 33.84
MKLAVQ 38.63
SQNTLKVQ 37.77
ALNLQDKQ 54.9
AKTIKITQ 55.72
MKGGKRVQ 38.76
Relative abundance
m/z

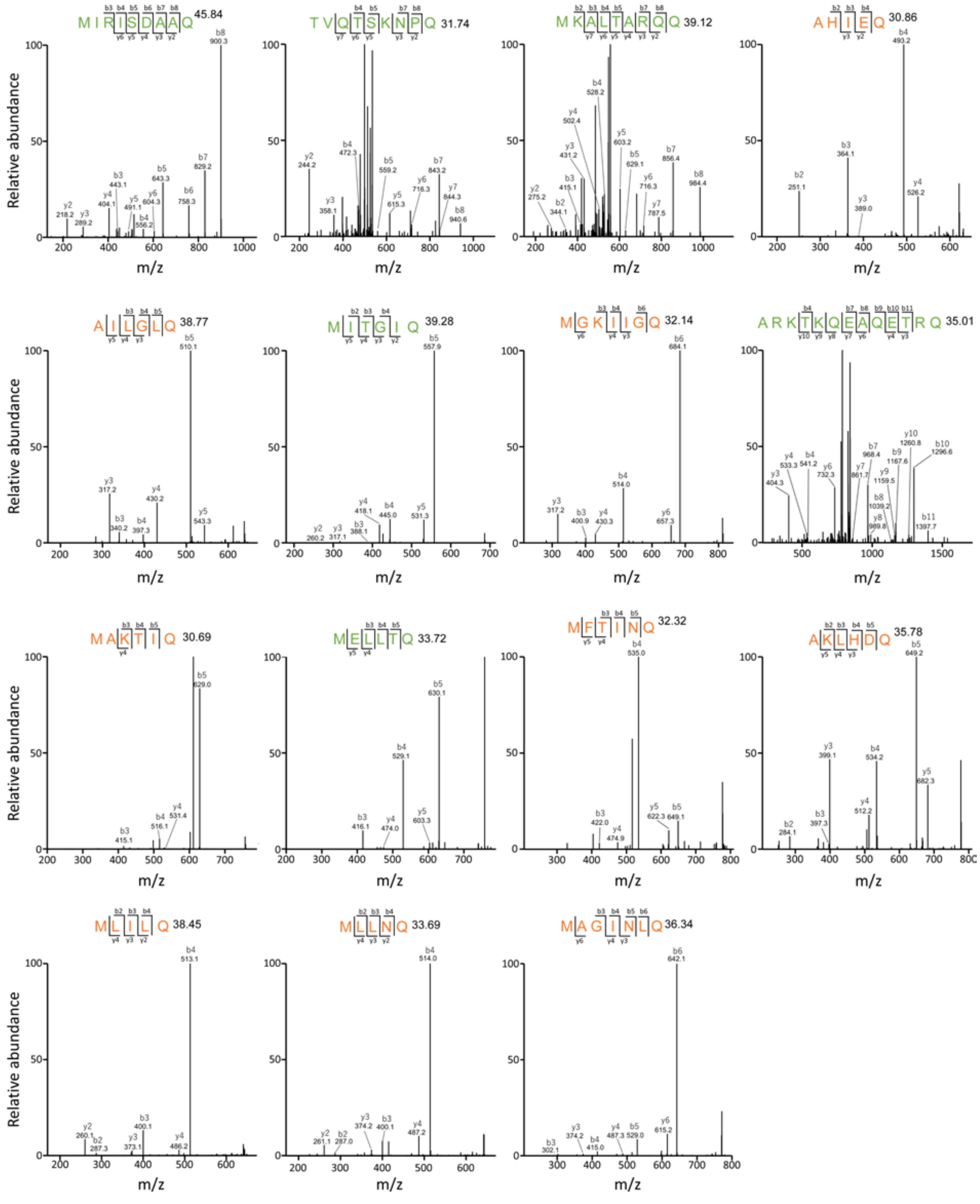

Supplementary Figure 8. MS sequencing of 55 nascent peptides of the isolated pep-tRNA^{Gln1}.

Each peptide with proton adduct was decomposed by CID. Sequence and peptide score calculated by MASCOT are indicated. Product ions are assigned to the sequence of the corresponding peptide. Nascent peptides derived from cognate and non-cognate pep-tRNAs are

shown in green and orange, respectively.

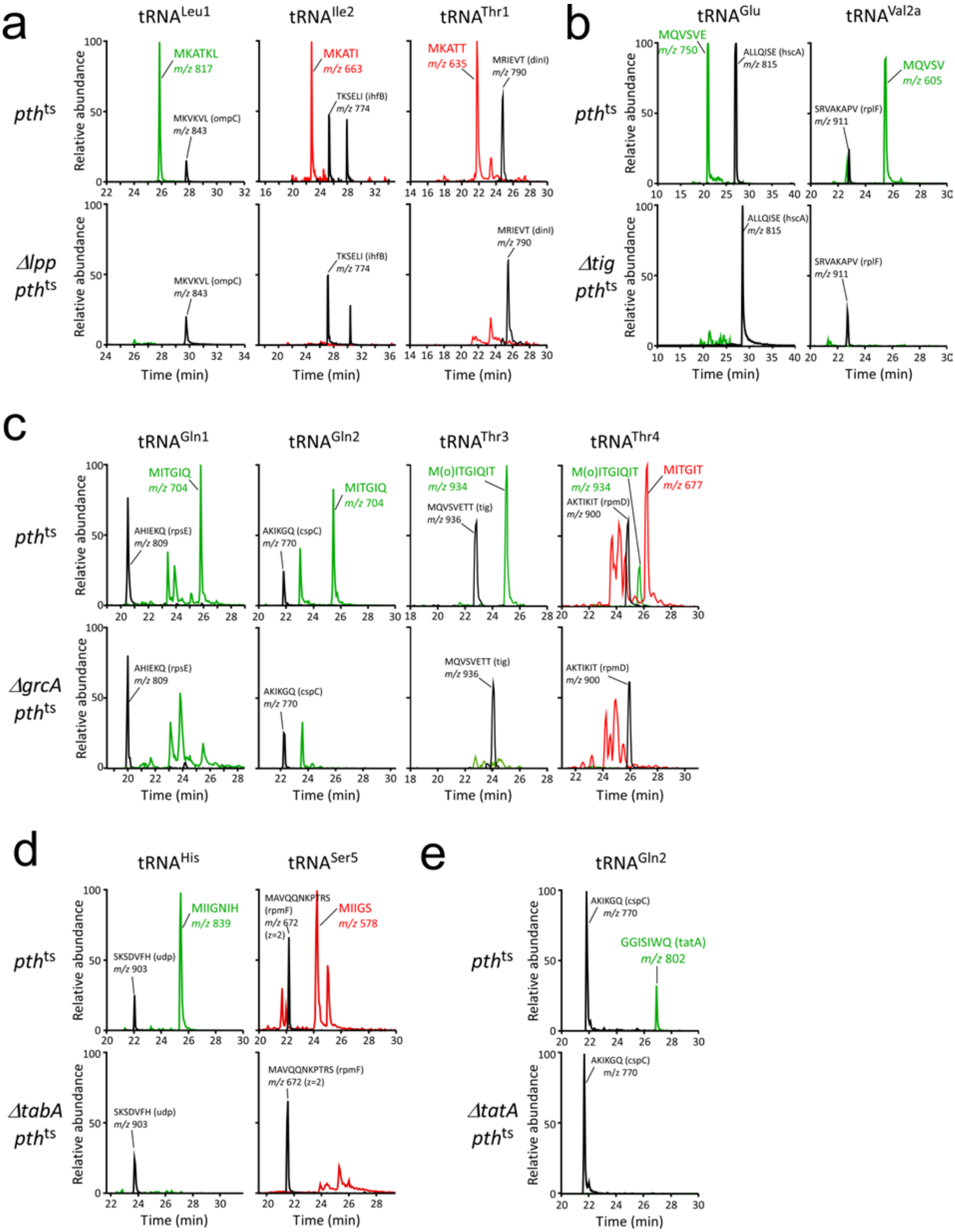

Supplementary Figure 9. Validation of the nascent peptides of the isolated pep-tRNAs. (a–e) Mass chromatograms for the nascent peptides derived from the cognate (green) and non-cognate (red) pep-tRNAs isolated from the *pth*ts (upper panels) and the respective knockouts

strain in the *pth*^{ts} background (lower panels). The sequence and *m/z* values of the peptide are indicated. Mass chromatograms of the nascent peptides derived from the other genes are shown in black as internal controls.

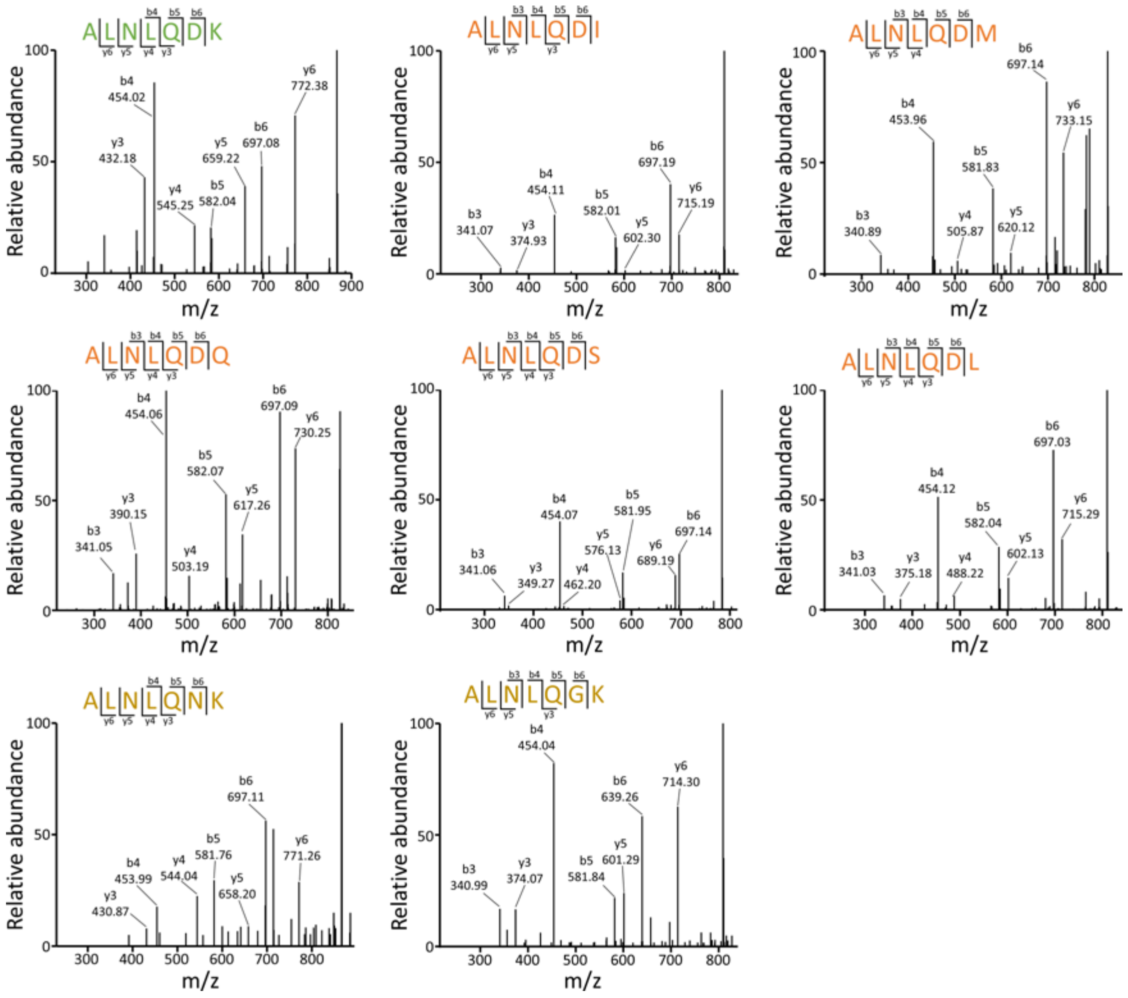

Supplementary Figure 10. MS sequencing of eight nascent peptides of isolated pep-tRNAs assigned to *rplJ* mRNA.

Each of eight peptides (shown in Figure 5e) with proton adduct was decomposed by CID. Product ions are assigned to the sequence of the corresponding peptide. Color codes for cognate, C_0X, and $C_{-1}X$ pep-tRNAs are shown in green, orange, and brown, respectively.

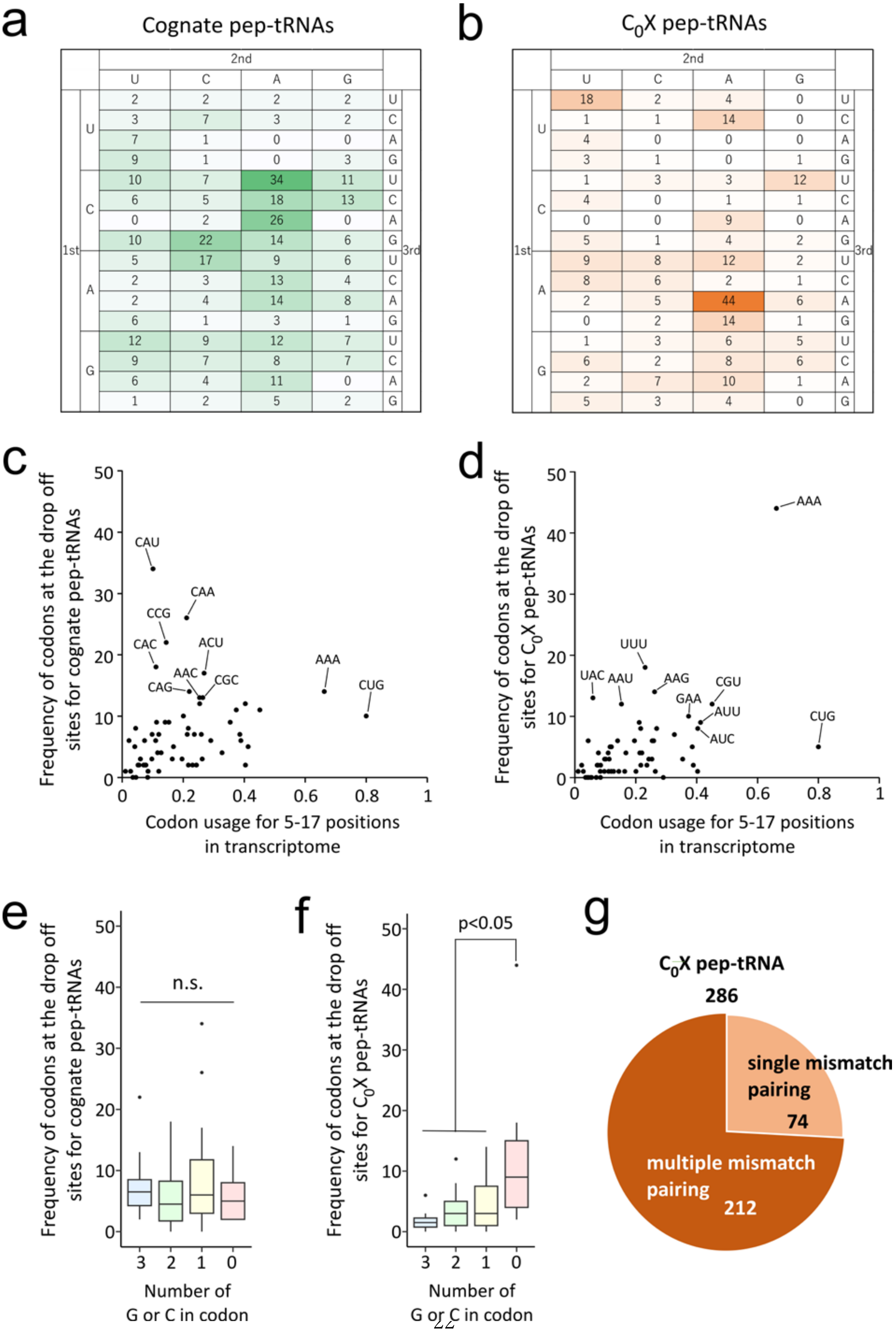
a
Cognate pep-tRNAs
b
C_0X pep-tRNAs
2nd
1st
3rd
c
Frequency of codons at the drop off sites for cognate pep-tRNAs
Codon usage for 5-17 positions in transcriptome
CAU
CAA
CCG
CAC
ACU
AAC
CAG
CGC
AAA
CUG
d
Frequency of codons at the drop off sites for C_0X pep-tRNAs
UUU
UAC
AAU
AAG
CGU
GAA
AUU
AUC
e
n.s.
Number of G or C in codon
f
p<0.05
g
C_0X pep-tRNA
286
single mismatch pairing
74
multiple mismatch pairing
212

Supplementary Figure 11. Characterization of codon frequency at the drop-off sites.

(a, b) Frequencies of codons at drop-off sites for cognate **(a)** and C_0X **(b)** pep-tRNAs. Level of codon frequency is depicted by the shade of green **(a)** and orange **(b)**, respectively.

(c, d) Correlation between codon usage for positions 5–17 in the transcriptome (x-axis) and the frequency of codons at drop-off sites (y-axis) for cognate pep-tRNAs **(c)** and C_0X pep-tRNAs **(d)**. The codon usage for positions 5–17 in the transcriptome is calculated based on the TPM value of each mRNA from the RNA-seq analyses of the pth^{ts} strain incubated at 43°C for 30 min.

(e, f) Boxplots of frequency of codons at the drop-off site for cognate pep-tRNA (n=8, 24, 22 and 7 for groups of 3, 2, 1 and 0 GCs, respectively) **(e)**, and C_0X pep-tRNA (n=8, 24, 22 and 7 for groups of 3, 2, 1 and 0 GCs, respectively) **(f)**. Codons are classified into four groups based on the number of G or C bases they contain. Boxplots show median (central line), upper and lower quartiles (box limits), maximum and minimum (whiskers). $p<0.05$ (two-sided Wilcoxon rank–sum test).

(g) Proportion of C_0X pep-tRNAs. Among 286 C_0X pep-tRNAs identified in the isolated pep-tRNA analyses, 74 cases have a single nucleotide mismatch, whereas 212 cases have multiple mismatches between the anticodon of the C_0X pep-tRNA and the drop-off site codon.

Source data are provided in Source Data file.

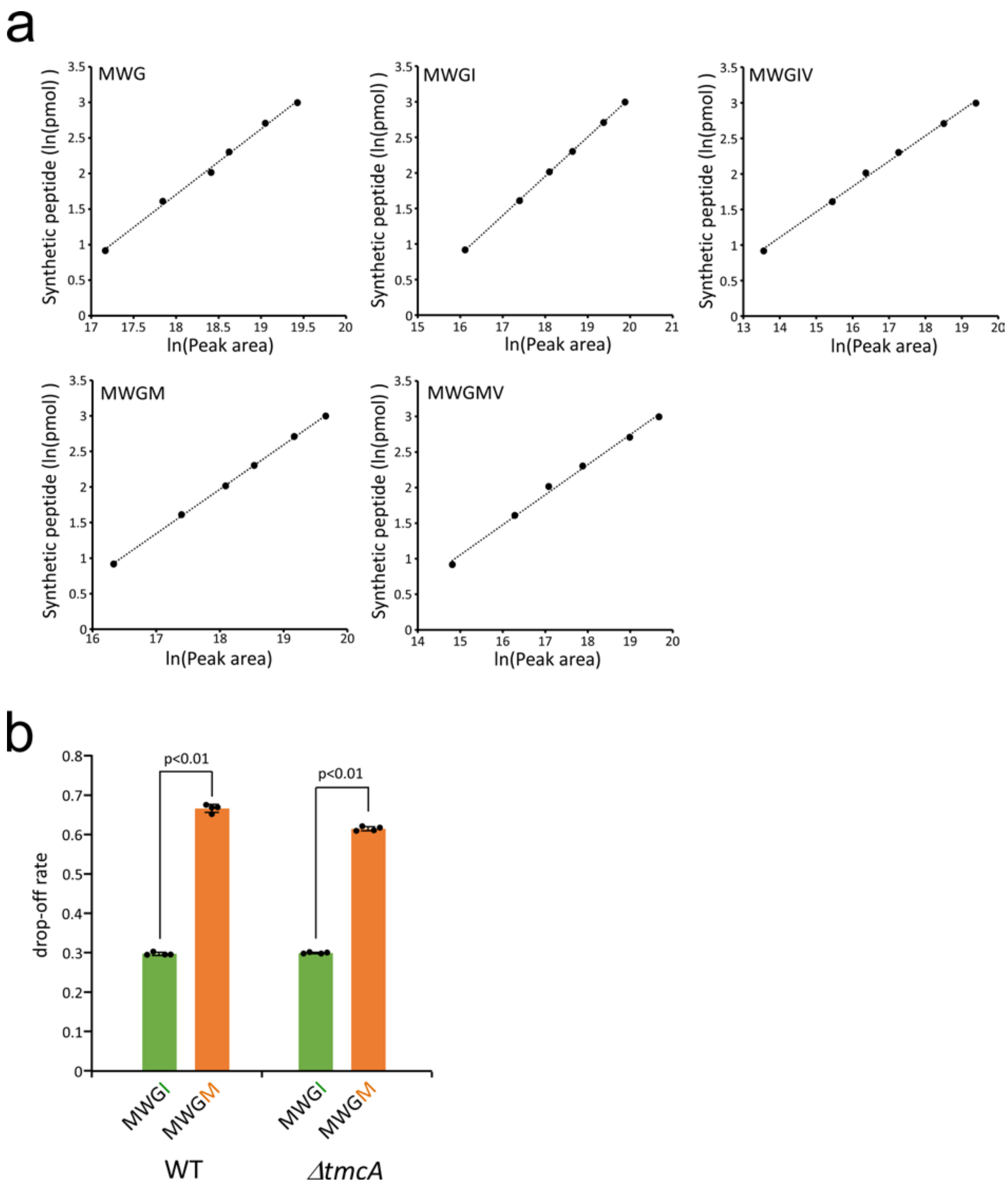

Supplementary Figure 12. Quantification and drop-off rate of pep-tRNAs dissociated from ribosome during translation of reporter mRNA

(a) Calibration curves of the synthetic acetyl peptides. *N*-acetylated oligo peptides for MWG, MWGI, MWGM, MWGIV, and MWGMV were subjected to PRC-MS at the indicated concentrations, and the mass was plotted versus the peak area of the respective mass chromatograms on a double logarithmic plot. The sequence of each peptide is shown in the corresponding panel.

(b) Drop-off rates of MWGI and MWGM pep-tRNAs in WT and *ΔtmcA* cells. The drop-off rate is calculated by dividing the amount of each tetrapeptide by the sum of amount of tetrapeptide and pentapeptide. Data are presented as means ± s.d. of four independent experiments. $p<0.01$ (two-tailed *t*-test). Exact p-values for WT and *ΔtmcA* are 6.6×10^{-10} and 3.8×10^{-11}, respectively. Source data are provided in Source Data file.

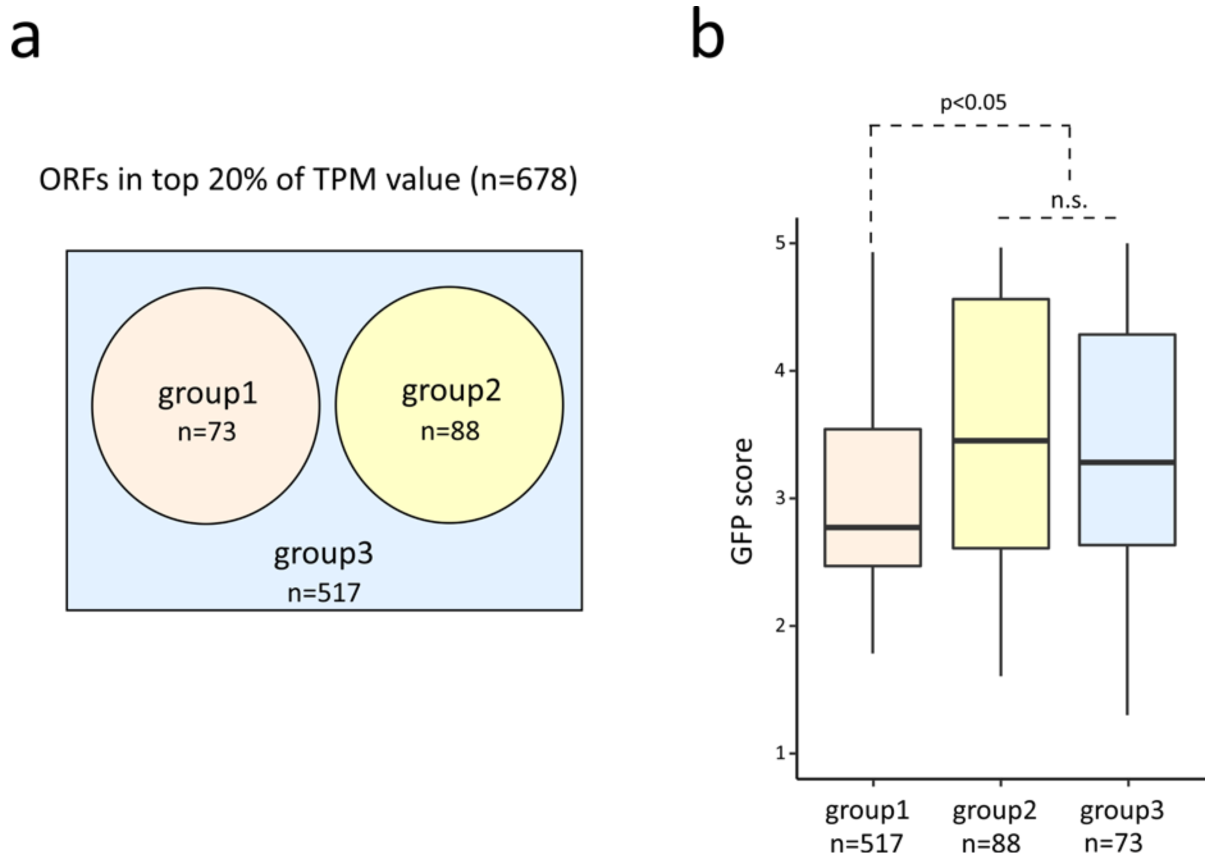

Supplementary Figure 13. Negative correlation between translation efficiency and pep-tRNA drop-off.

(a) Venn diagram showing the grouping of highly expressed *E. coli* genes (678 ORFs) with top 20% of TPMs from our RNA-seq data into each group. Group 1 has 73 ORFs in which pep-tRNAs are dissociated from 5th and 6th codon positions. Group 2 has 88 ORFs in which pep-tRNAs are dissociated from 7th codon or later positions. Group 3 has 517 ORFs in which no pep-tRNAs are detected.

(b) Boxplots of GFP scores [2] corresponding to the 3rd to 5th codon positions of *E. coli* ORFs of each group. Boxplots show median (central line), upper and lower quartiles (box limits), maximum and minimum (whiskers). $p<0.05$ (two-sided Wilcoxon rank–sum test)

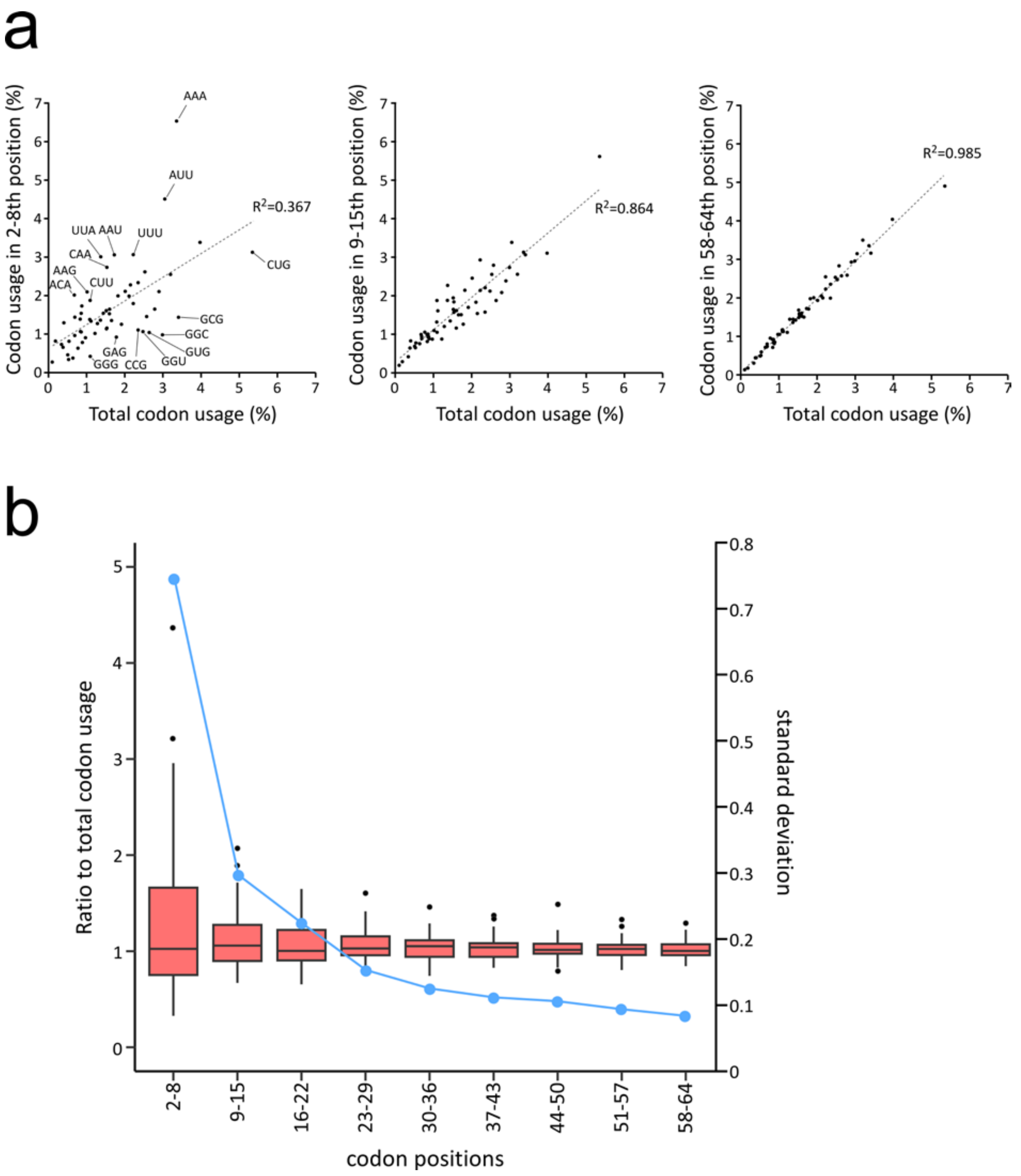

Supplementary Figure 14. Codon usage deviation observed in the beginning of *E. coli* genes.

(a) Correlation between overall codon usage and codon usage in the 2–8th positions (left panel), the 9–15th positions (middle panel) and the 58–64th positions (right panel) of 4,143 protein coding genes (ORFs) from *E. coli* K-12 MG1655 U00096.3 Database table from EcoGene (http://ecogene.org/).

(b) Codon usage ratio to the total codon usage in the indicated positions of *E. coli* ORFs are

box-plotted (n=61 for all box plots). Boxplots show median (central line), upper and lower quartiles (box limits), maximum and minimum (whiskers). Standard deviation (σ) of each box plot is calculated and plotted as blue circle. Source data are provided in Source Data file.

Supplementary references

1. Saito, K., Green, R. & Buskirk, A.R. Ribosome recycling is not critical for translational coupling in Escherichia coli. *Elife* **9**(2020).
2. Verma, M. et al. A short translational ramp determines the efficiency of protein synthesis. *Nat Commun* **10**, 5774 (2019).
