# Supplementary material for: Quality control of protein synthesis in the early elongation stage
Source: Nat Commun. 2023 May 17;14:2704. doi: 10.1038/s41467-023-38077-5 (PMC10192219; doi:10.1038/s41467-023-38077-5)
Supplement: Supplementary file 3 — Description of Additional Supplementary Files [file 41467_2023_38077_MOESM3_ESM.pdf]

Description of Additional Supplementary Files

File Name: Supplementary Data 1
Description: Nascent peptides derived from pep-tRNAs detected in this study.

File Name: Supplementary Data 2
Description: GFP scores of highly expressed 678 ORFs and grouping of them based on pep-tRNA drop-off.

File Name: Supplementary Data 3
Description: Frequency of each amino acid in N-terminal region (2nd-10th codon region) of E. coli ORFs and in the identified pep-tRNAs in this study.

File Name: Supplementary Data 4
Description: List of probes, primers and strains used in this study.
