# Supplementary material for: Quality control of protein synthesis in the early elongation stage
Source: Nat Commun. 2023 May 17;14:2704. doi: 10.1038/s41467-023-38077-5 (PMC10192219; doi:10.1038/s41467-023-38077-5)
Supplement: Supplementary file 8 — Reporting Summary [file 41467_2023_38077_MOESM8_ESM.pdf]

nature portfolio

Corresponding author(s): Tsutomu Suzuki, Asuteka Nagao

Last updated by author(s): Mar 31, 2023

Reporting Summary

Nature Portfolio wishes to improve the reproducibility of the work that we publish. This form provides structure for consistency and transparency in reporting. For further information on Nature Portfolio policies, see our Editorial Policies and the Editorial Policy Checklist.

Statistics

For all statistical analyses, confirm that the following items are present in the figure legend, table legend, main text, or Methods section.

n/a	Confirmed	
☐	☒	The exact sample size (*n*) for each experimental group/condition, given as a discrete number and unit of measurement
☐	☒	A statement on whether measurements were taken from distinct samples or whether the same sample was measured repeatedly
☐	☒	The statistical test(s) used AND whether they are one- or two-sided<br>*Only common tests should be described solely by name; describe more complex techniques in the Methods section.*
☒	☐	A description of all covariates tested
☒	☐	A description of any assumptions or corrections, such as tests of normality and adjustment for multiple comparisons
☐	☒	A full description of the statistical parameters including central tendency (e.g. means) or other basic estimates (e.g. regression coefficient) AND variation (e.g. standard deviation) or associated estimates of uncertainty (e.g. confidence intervals)
☐	☒	For null hypothesis testing, the test statistic (e.g. *F*, *t*, *r*) with confidence intervals, effect sizes, degrees of freedom and *P* value noted<br>*Give P values as exact values whenever suitable.*
☒	☐	For Bayesian analysis, information on the choice of priors and Markov chain Monte Carlo settings
☒	☐	For hierarchical and complex designs, identification of the appropriate level for tests and full reporting of outcomes
☒	☐	Estimates of effect sizes (e.g. Cohen's *d*, Pearson's *r*), indicating how they were calculated

Our web collection on statistics for biologists contains articles on many of the points above.

Software and code

Policy information about availability of computer code

Data collection	The photographs of the LB plates were taken with a digital camera and saved as jpeg files.<br>The gel images were obtained by FLA-7000.<br>The RNA-Seq raw data was obtained by HiSeq 4000 (50-bp, single-read).<br>The data of ribosome profiling was obtained by HiSeq 3000 (100-bp, pair-read)<br>LC/MS data was obtained by LTQ Orbitrap XL, Q Exactive Hybrid Quadrupole-Orbitrap Mass Spectrometer, Orbitrap Eclipse and LCQ-Advantage Ion-trap Mass Spectrometer.
Data analysis	Canvas 11, R(4.0.2), Microsoft Excel (2016), GraphPad prism9, ACD/ChemSketch(Freeware, 2018.2.1) and ChemDraw20.1 were used to draw figures and analyze statistical data.<br>Multi Gauge3.0 was used for gel images.<br>fastp was used for raw read quality control and adapter trimming.<br>Trimmed reads were mapped against Escherichia coli genome reference using bowtie2.<br>Mapped counts were determined by Ribo-Seq tools (https://github.com/ingolia-lab/RiboSeq).<br>Xcalibur4.5 SP1, MS-Dial4.9 and MASCOT Daemon (2.6.0) were used for mass spec analysis.

For manuscripts utilizing custom algorithms or software that are central to the research but not yet described in published literature, software must be made available to editors and reviewers. We strongly encourage code deposition in a community repository (e.g. GitHub). See the Nature Portfolio guidelines for submitting code & software for further information.

Data

Policy information about availability of data

All manuscripts must include a data availability statement. This statement should provide the following information, where applicable:

- Accession codes, unique identifiers, or web links for publicly available datasets
- A description of any restrictions on data availability
- For clinical datasets or third party data, please ensure that the statement adheres to our policy

All data that support the findings of this study are provided in the article, Supplementary Information or Source data file. Ribosome profiling and RNA-seq data are deposited in DDBJ (accession numbers: DRA012800[https://ddbj.nig.ac.jp/resource/sra-submission/DRA012800], DRA012753[https://ddbj.nig.ac.jp/resource/sra-submission/DRA012753]). The mass spectrometry proteomics data have been deposited to the ProteomeXchange Consortium via the PRIDE partner repository with the dataset identifiers PXD030805[https://www.doi.org/10.6019/PXD030805], PXD030806[https://www.doi.org/10.6019/PXD030806], PXD030807[https://www.doi.org/10.6019/PXD030807] and PXD030808[https://www.doi.org/10.6019/PXD030808]. E. coli genome reference used for RNA-seq and Ribosome profiling analyses is NC_000913.2. Source data are provided with this paper.

Human research participants

Policy information about studies involving human research participants and Sex and Gender in Research.

Reporting on sex and gender	This study is not applicable because it did not involve human research.
Population characteristics	This study is not applicable because it did not involve human research.
Recruitment	This study is not applicable because it did not involve human research.
Ethics oversight	This study is not applicable because it did not involve human research.

Note that full information on the approval of the study protocol must also be provided in the manuscript.

Field-specific reporting

Please select the one below that is the best fit for your research. If you are not sure, read the appropriate sections before making your selection.

☒ Life sciences ☐ Behavioural & social sciences ☐ Ecological, evolutionary & environmental sciences

For a reference copy of the document with all sections, see nature.com/documents/nr-reporting-summary-flat.pdf

Life sciences study design

All studies must disclose on these points even when the disclosure is negative.

Sample size	Sample size of each experiment was described in the figure legends of the main text and supplementary information. Each sample size was determined according to to previous studies in our laboratory or published papers performing similar experiments. No statistical method was used to pre-determine each sample size.
Data exclusions	No data were excluded.
Replication	Experiments in the growth analyses of various strains, the analyses on accumulation level of dipep-tRNAs with various concentrations of Tyl, the analyses on the overall change in aa-tRNAs and dipep-tRNAs in the pthts strain that expresses different levels of RRF, the isolation of individual pep-tRNAs, the analyses of [18O]-labeled peptides from pep-tRNA, the validation of the pep-tRNA using knockout strains and the construction of calibration curves for quantification of pep-tRNAs from reporter gene were performed one time with no replication. The other experiments were replicated in at least two biologically independent samples or independently performed at least two times with the same results.
Randomization	Randomization was not applicable in this study because there is no statistic test that requires randomization of samples.
Blinding	Blinding was not required in this study because the results of measurement or analysis was not affected by knowledge of sample identities.

Reporting for specific materials, systems and methods

We require information from authors about some types of materials, experimental systems and methods used in many studies. Here, indicate whether each material, system or method listed is relevant to your study. If you are not sure if a list item applies to your research, read the appropriate section before selecting a response.

Materials & experimental systems

n/a	Involved in the study
☒	☐ Antibodies
☒	☐ Eukaryotic cell lines
☒	☐ Palaeontology and archaeology
☒	☐ Animals and other organisms
☒	☐ Clinical data
☒	☐ Dual use research of concern

Methods

n/a	Involved in the study
☒	☐ ChIP-seq
☒	☐ Flow cytometry
☒	☐ MRI-based neuroimaging
